# Generalized Tetanus in a Four-Month-Old Labrador Retriever: Complete Recovery and One-Year Follow-Up in a Rare Canine Case

**DOI:** 10.3390/life16101592

**Published:** 2026-09-23

**Authors:** Cristina Fernoagă, Raluca Mihaela Turbatu, Alexandru Gabriel Neagu, Ruxandra Pavel, Niculae Tudor, Mihai Musteata

**Affiliations:** 1 Faculty of Veterinary Medicine, University of Agronomic Sciences and Veterinary Medicine of Bucharest, 011464 Bucharest, Romania; cristina.fernoaga@fmvb.usamv.ro (C.F.); alexandru.neagu@fmvb.usamv.ro (A.G.N.); ruxandra.pavel@fmvb.usamv.ro (R.P.);; 2 Faculty of Veterinary Medicine, University of Life Sciences “Ion Ionescu de la Brad”, 700490 Iasi, Romania

**Keywords:** tetanus, *Clostridium tetani*, tetanospasmin, dog, puppy, generalized rigidity

## Abstract

Tetanus is an infrequent, potentially fatal neurological disease in dogs, caused by tetanospasmin, the neurotoxin of *Clostridium tetani*, which reaches the central nervous system by retrograde axonal transport and blocks the release of the inhibitory neurotransmitters glycine and γ-aminobutyric acid. Dogs are comparatively resistant to the toxin, and canine reports remain scarce. This report describes generalized tetanus in a four-month-old, 13 kg male Labrador Retriever presented with an axillary wound sustained approximately one month earlier and neurological signs progressing over approximately seven days, from gait disturbance to generalized whole-body rigidity. On examination the dog showed trismus, risus sardonicus, cervical hyperextension, opisthotonos, third-eyelid protrusion, a sawhorse stance, hyperthermia and intermittent respiratory distress. The diagnosis was established clinically, following a structured neurological work-up and exclusion of the principal differentials, as confirmatory assays were unavailable. Total serum calcium was within the reference interval; serum creatine kinase was elevated; and venous blood-gas analysis indicated a mild, compensated metabolic acidosis. Management comprised metronidazole and clindamycin, equine tetanus antitoxin, diazepam, meloxicam and intensive nursing care in a low-stimulation environment. The dog improved from the first day of hospitalization and recovered without residual deficits, with no recurrence over one year of follow-up. The favorable outcome, in a young canine patient for which the literature would predict a guarded prognosis, is consistent with the absence of clinically detectable autonomic and cardiorespiratory dysfunction and with meticulous supportive care.

## 1. Introduction

Tetanus is caused by tetanospasmin, a metalloprotease neurotoxin produced by the anaerobic, spore-forming bacterium *Clostridium tetani* under the low-oxygen conditions of a contaminated wound. After local production, the toxin is internalized at the neuromuscular junction and transported retrogradely along motor axons to the spinal cord and brainstem, where it cleaves synaptobrevin and blocks the presynaptic release of the inhibitory neurotransmitters glycine and γ-aminobutyric acid (GABA); the resulting loss of inhibition of the α-motor neuron produces the sustained muscular rigidity and spasms that define the disease [1]. Although the pathophysiology is best characterized in human medicine, in which tetanus remains an important cause of preventable death and is graded by clinical severity, the same mechanism operates across species [2].

Among domestic animals, susceptibility to tetanospasmin varies widely: horses and humans are highly susceptible, whereas dogs are comparatively resistant, which is reflected in the low frequency of canine reports [3]. Consequently, the clinical experience with canine tetanus derives largely from single cases and from a small number of retrospective series [3,4,5,6,7]. In dogs, tetanus may present in the localized form, restricted to the region of the wound, or in the generalized form, characterized by trismus, risus sardonicus, extensor rigidity of the limbs, opisthotonos and protrusion of the third eyelid [3,4]. Reported case fatality is substantial, and the outcome is determined chiefly by the development of respiratory and autonomic complications rather than by the muscular rigidity itself [4,5].

Reports of canine tetanus in very young dogs are rare, and the available series indicate that youth is a marker of clinical severity rather than of a benign course [4,5]. The present case is described in detail to inform prognostic expectations in this under-represented age group and to reinforce the recognition of tetanus among the differential diagnoses of acute generalized neuromuscular rigidity in dogs.

## 2. Case Presentation

### 2.1. Signalment and History

A four-month-old, unneutered male Labrador Retriever weighing 13 kg was presented to the Veterinary Teaching Hospital with generalized stiffness and rigidity affecting the whole body, most evident in the neck and face. The dog was unable to walk unaided and could be moved only when lifted by the owner, exhibiting whole-body muscular rigidity and an uncoordinated gait (Figure 1). The owner reported that the dog lived outdoors in a community of animals and that, approximately one month earlier, a wound had been present in the axillary region. Neurological signs had developed approximately seven days before presentation, beginning with gait disturbance and progressing to generalized rigidity; trismus and difficulty with prehension were present at admission, although their exact onset could not be dated by the owner; and respiratory signs were first documented at admission. Appetite had decreased because stiffness of the jaw prevented normal prehension and mastication, and the dog’s vocalization was altered such that it was unable to bark. Symptomatic treatment (antibiotics and anti-inflammatory drugs) had been administered elsewhere for two days without improvement. The dog had not been vaccinated and had previously tested negative for canine distemper.

### 2.2. Physical Examination

On examination the following abnormalities were recorded: hyperthermia (40.5 °C); intermittent respiratory distress and tachypnea (46 breaths per minute) attributed to spasm of the respiratory musculature; a normal heart rate (104 beats/min at initial evaluation and 112 beats/min at the subsequent cardiological examination), with no cardiac murmurs or other abnormal heart sounds on auscultation; a healed scar approximately 0.5 cm in diameter in the left axillary region, consistent with the presumed portal of entry; and generalized muscular stiffness with a characteristic sawhorse stance, the pelvic limbs being more severely affected than the thoracic limbs (Figure 2 and Figure 3). The dog was able to eat and drink only with assistance, and the deciduous dentition was in the process of being replaced. Trismus, together with contracture of the facial, parietal and temporal muscles, produced the characteristic risus sardonicus, and the contractions were accentuated by auditory and visual stimuli. Protrusion of the third eyelid was present bilaterally. The mucous membranes were pink with a capillary refill time under three seconds, and deep abdominal palpation was hindered by the tense abdominal musculature. Systolic arterial blood pressure, measured by Doppler sphygmomanometry, was 130 mmHg.

### 2.3. Neurological Examination

Mental status was normal. Posture was dominated by muscular rigidity of all four limbs, cervical hyperextension and a broad-based stance; in lateral recumbency the dog showed extensor rigidity of all limbs, opisthotonos and an inability to change position, which was maintained during attempted ambulation. During the first 48 h the dog was unable to rise unaided; when assisted, it could maintain a standing position and walk. Examination of the cranial nerves revealed bilateral miosis with prominent third eyelids and prompt, symmetrical pupillary light reflexes; contracture of the parietal and temporo-auricular musculature with trismus and risus sardonicus; and difficulty swallowing. On assessment of the spinal reflexes, the patellar reflex was difficult to elicit due to the extensor rigidity, and limb flexion was achieved only with considerable effort. Muscle tone was markedly and diffusely increased in all four limbs, and postural reactions could not be reliably assessed owing to the generalized rigidity. No seizure activity was observed at any time.

### 2.4. Diagnostic Investigations

A complete neurological evaluation was performed following a structured diagnostic approach integrating history, clinical findings, neurological examination, and neuroanatomical assessment to guide the differential diagnosis [8,9]. Potential causes of generalized neuromuscular rigidity were subsequently considered according to the principal etiological categories, including toxic, inflammatory/infectious, metabolic, and neoplastic disorders. Given the history of a wound sustained approximately one month previously, the characteristic clinical signs and the rapid progression of the illness, the most likely diagnosis was considered to be tetanus, caused by the tetanospasmin toxin. Other hypotheses, such as strychnine poisoning or spasms caused by calcium deficiency (hypocalcemic tetany), were considered less likely: There was no known exposure to toxic baits. The clinical picture consisted of sustained rigidity with trismus and risus sardonicus rather than the intermittent, stimulus-triggered tetanic seizures typical of strychnine intoxication, and total serum calcium was within the reference interval (Table 1). Because inoculation of patient serum into laboratory animals is slow and not routinely available and because neither toxin detection nor antibody titers could be obtained, the diagnosis was established clinically, in keeping with the diagnostic standard applied in the published canine series [3,4,6].

A complete blood count, serum biochemistry, and venous blood-gas and electrolyte analyses were performed using the IDEXX ProCyte Dx Hematology Analyzer, IDEXX Catalyst Dx Chemistry Analyzer, and IDEXX VetStat Electrolyte and Blood-Gas Analyzer, respectively (IDEXX Laboratories, Inc., Westbrook, ME, USA). Abdominal ultrasonography was performed using a Vetus 9 ultrasound system (Mindray, Shenzhen, China), while the cardiological examination included echocardiographic evaluation using a MyLab™ X7 ultrasound system (Esaote, Genoa, Italy), to support the clinical impression and screen for systemic complications. Echocardiographic examination revealed no abnormalities of the measured parameters; continuous electrocardiographic monitoring was not performed. The complete results are presented in Table 1. The principal abnormality was an increased serum creatine kinase activity (990 U/L; upper reference limit 436 U/L). Venous blood-gas analysis showed a normal pH (7.37) with mildly decreased bicarbonate (19.7 mmol/L) and total carbon dioxide (20.8 mmol/L) and an anion gap of 22 mmol/L, consistent with a mild, compensated metabolic acidosis. Total serum calcium (10.0 mg/dL) and the remaining electrolytes were within their reference intervals, and erythroid values slightly below the adult reference intervals were considered consistent with the physiological hematology of a growing puppy [10]. The mildly decreased urea nitrogen and the marginally increased gamma-glutamyl transferase activity were considered clinically insignificant. Elevated creatine kinase is a recognized, though non-specific, finding in tetanus and is attributable to skeletal-muscle injury resulting from sustained neuromuscular hyperactivity [4,6].

### 2.5. Treatment and Management

Antimicrobial therapy comprised metronidazole (Metrobactin^®^, Dechra Regulatory B.V., Bladel, The Netherlands; 10 mg/kg every 12 h) and, because the presumed portal of entry was more than one month old, clindamycin (Zodon^®^, Ceva Santé Animale, Libourne, France; 11 mg/kg/day). Gastrointestinal adverse effects were managed with a proton-pump inhibitor (Pantoprazole Terapia^®^ 20 mg, Terapia S.A., Cluj-Napoca, Romania; 1 mg/kg/day). A hepatoprotective supplement (Hepatiale^®^ Forte 170 mg, VetExpert/Vet Planet Sp. z o.o., Łomianki, Poland; one capsule every 12 h) and a probiotic (Synbiotic^®^ D-C, ADM Protexin Ltd., South Petherton, Somerset, UK; one capsule/day) were administered to mitigate potential adverse effects associated with antimicrobial therapy. Gastrointestinal motility was monitored ultrasonographically and required no additional treatment.

Equine tetanus antitoxin (SER CLOSTETAN^®^, 300 international units (IU)/mL, Bioveta a.s., Ivanovice na Hané, Czech Republic) was administered at 250 IU/kg/day, subcutaneously, for three consecutive days, following a prior intradermal sensitivity test, with the aim of neutralizing circulating, unbound toxin. Muscular rigidity and spasms were controlled with diazepam (Diazepam Terapia^®^ 5 mg/mL solution for injection, Terapia S.A., Cluj-Napoca, Romania; 0.25 mg/kg intravenously every 12 h for seven days), while meloxicam (Metacam^®^, 5 mg/mL solution for injection, Boehringer Ingelheim Vetmedica GmbH, Ingelheim am Rhein, Germany; 0.2 mg/kg every 24 h) was administered as anti-inflammatory therapy.

Supportive care comprised lactated Ringer’s solution (B. Braun^®^ Melsungen AG, Melsungen, Germany; 3 mL/kg/h), assisted feeding, ocular lubrication, oral hygiene, and continuous monitoring in a calm environment with minimal auditory and visual stimulation. Short periods of oxygen supplementation were employed during two brief episodes of respiratory distress.

### 2.6. Outcome and Follow-Up

The dog was hospitalized for seven days. Heart rate was monitored daily and remained within physiological limits, with no arrhythmias detected on auscultation. Body temperature, 40.5 °C at admission, decreased to 38.7–39.4 °C within the first 24 h and remained between 38.5 and 39.0 °C from the third day onwards. From the first day of hospitalization the clinical course was favorable: the generalized muscular contracture progressively resolved, and the dog regained the ability to eat and to walk independently, with recovery of phonation during hospitalization. At the end of the first week, given the favorable evolution, the dog was discharged with a recommendation to continue supportive care for a further seven days and recovered without residual neurological deficits. Scheduled re-examinations (14 days after discharge, monthly for three months, then every three months for one year), together with visits for unrelated routine care, each included a complete clinical and neurological examination; no recurrence, residual neurological deficits or abnormalities attributable to the tetanus episode were identified at any point.

## 3. Discussion

This report describes generalized tetanus in a four-month-old Labrador Retriever that achieved complete clinical recovery, representing a rarely documented presentation in a very young dog and adding to the limited literature on canine tetanus in Romania. The clinical presentation—trismus, risus sardonicus, cervical hyperextension, opisthotonos, third-eyelid protrusion and generalized extensor rigidity—is characteristic of generalized tetanus and reflects the loss of inhibitory neurotransmission produced by tetanospasmin at the level of the central nervous system [1,3]. Although the portal of entry is peripheral and the clinical picture is dominated by neuromuscular signs, the functional lesion is central, which accounts for the sustained agonist–antagonist co-contraction observed in this patient [1]. This mechanism also provides an explanation for the apparent bilateral loss of the patellar reflex: rather than indicating a lower-motor-neuron lesion, the difficulty in eliciting the myotatic reflex is consistent with the sustained co-contraction of antagonistic muscle groups that mechanically constrains the phasic response, although, in the absence of electrodiagnostic testing, this interpretation remains presumptive. The transient loss of the ability to bark, with recovery of phonation as the rigidity subsided, may likewise be attributed to tetanic involvement of the laryngeal and pharyngeal musculature.

Two features of the presentation merit emphasis. First, according to the four-grade clinical severity classification described by Burkitt et al. [4], in which Grade III comprises Grade I/II signs together with recumbency and tetanic spasms or seizures, and Grade IV additionally requires abnormal heart rate or rhythm, arterial blood pressure or respiration, the findings at admission (generalized rigidity with trismus, dysphagia, stimulus-accentuated tetanic spasms and, during the first 48 h, inability to rise unaided) corresponded to Grade III. The intermittent tachypnea was attributed to spasm of the respiratory musculature rather than to central respiratory dysregulation, and heart rate, rhythm and arterial blood pressure remained normal, so the case was not classified as Grade IV. Second, the young age of the patient is noteworthy. In the two largest canine series [4,5], younger age has been associated with more severe disease and with a poorer outcome. Burkitt et al. reported that younger dogs were more likely to develop severe clinical signs [4]. Furthermore, Zitzl et al. found that non-survivors were significantly more often younger than two years of age [5]. Complete recovery in a four-month-old dog with a generalized presentation therefore represents a notable exception to this reported age-related risk and complements the favorable outcome recently documented in another very young dog with Grade III disease [11]. The approximately three-week interval between the wound and the onset of neurological signs lies within the upper range of incubation periods reported in canine series [3,4,6], and the subsequent progression over approximately seven days is consistent with the described course of generalized disease.

The favorable outcome is best interpreted in light of the prognostic determinants identified in the canine literature. Burkitt et al. concluded that the prognosis for survival is good provided that abnormalities in heart rate or blood pressure do not develop [4], and Zitzl et al. found respiratory complications to be significantly associated with non-survival, with none of the dogs reaching the autonomic-dysfunction category surviving [5]. In the present case, no clinically detectable autonomic dysfunction developed: heart rate remained within physiological limits on daily monitoring, without arrhythmias, and the hyperthermia at admission resolved within the first 48 h without recurrence. Aspiration pneumonia, the complication most strongly linked to death in these series, did not develop, and the two brief episodes of respiratory distress were managed with short periods of oxygen supplementation. The clinical course is thus consistent with the pattern in which severe but autonomically stable tetanus carries a favorable prognosis when supportive care is meticulous. This relationship parallels the human experience, in which autonomic dysfunction is a principal determinant of mortality [2,12].

The pharmacological management was consistent with current recommendations for the disease. Metronidazole was selected as the primary antimicrobial agent; evidence-based reviews support metronidazole over penicillin, the latter being a GABA antagonist that may theoretically aggravate the disinhibition caused by tetanospasmin [7], and metronidazole is the antibiotic most frequently reported in recent canine series [6]. Clindamycin was added to broaden anaerobic coverage because the presumed portal of entry was old and already healed at presentation, precluding surgical debridement; for the same reason, bacterial culture of the wound was not attempted, as the healed scar offered no material for sampling. Muscular rigidity was controlled with the benzodiazepine diazepam, which restores inhibitory tone through potentiation of GABA-A receptor function and is the muscle-relaxant class supported by systematic review in human tetanus [13], combined with nursing care that minimized sensory stimulation. Tetanus antitoxin was administered, after an intradermal sensitivity test, to neutralize unbound circulating toxin; antitoxin cannot reverse toxin already internalized at the synapse, and its use in dogs is not fully standardized, but it is administered in the majority of reported canine cases with the aim of limiting further progression [3,6].

This case report has certain limitations, the main one being the inability to confirm the diagnosis through toxin or antibody detection, as these tests were unavailable. However, the diagnosis was based on correlating the wound history with the pathognomonic clinical signs and excluding other similar conditions, an approach that reflects the clinical standard applied in the literature regarding canine cases [3,4,6]. A further limitation is inherent to the single-patient nature of the report, which precludes any generalizable conclusion regarding treatment efficacy; the detailed documentation of the clinical course does, nonetheless, adds to the still-limited body of evidence on tetanus in young dogs [4,5]. In addition, serum magnesium and aspartate aminotransferase were not measured, and, because base excess and lactate were not determined, the acid–base status was characterized from pH, bicarbonate, carbon dioxide and the anion gap alone. Blood pressure was measured only at admission, and continuous electrocardiographic monitoring was not available; autonomic surveillance therefore relied on daily clinical assessment of heart rate, cardiac auscultation and body temperature (measured several times daily during the first two days and once daily thereafter), and subclinical autonomic disturbances cannot be formally excluded. Finally, although clinical and neurological follow-up extended over one year without evidence of recurrence or residual deficits, electrodiagnostic examination was not performed during this period, and subtle subclinical abnormalities cannot therefore be formally excluded.

## 4. Conclusions

This report documents the successful management of severe generalized tetanus in a four-month-old dog, a subgroup in which the literature would predict a guarded prognosis. The favorable outcome, achieved through structured clinical diagnosis, appropriate antimicrobial and antitoxin therapy and intensive, autonomically focused supportive care, is consistent with published evidence that canine tetanus carries a favorable prognosis in the absence of clinically detectable cardiorespiratory and autonomic dysfunction [4,5]. Tetanus should be considered in the differential diagnosis of any dog presenting with generalized neuromuscular rigidity and a history of a penetrating wound.

## Figures and Tables

**Figure 1:**
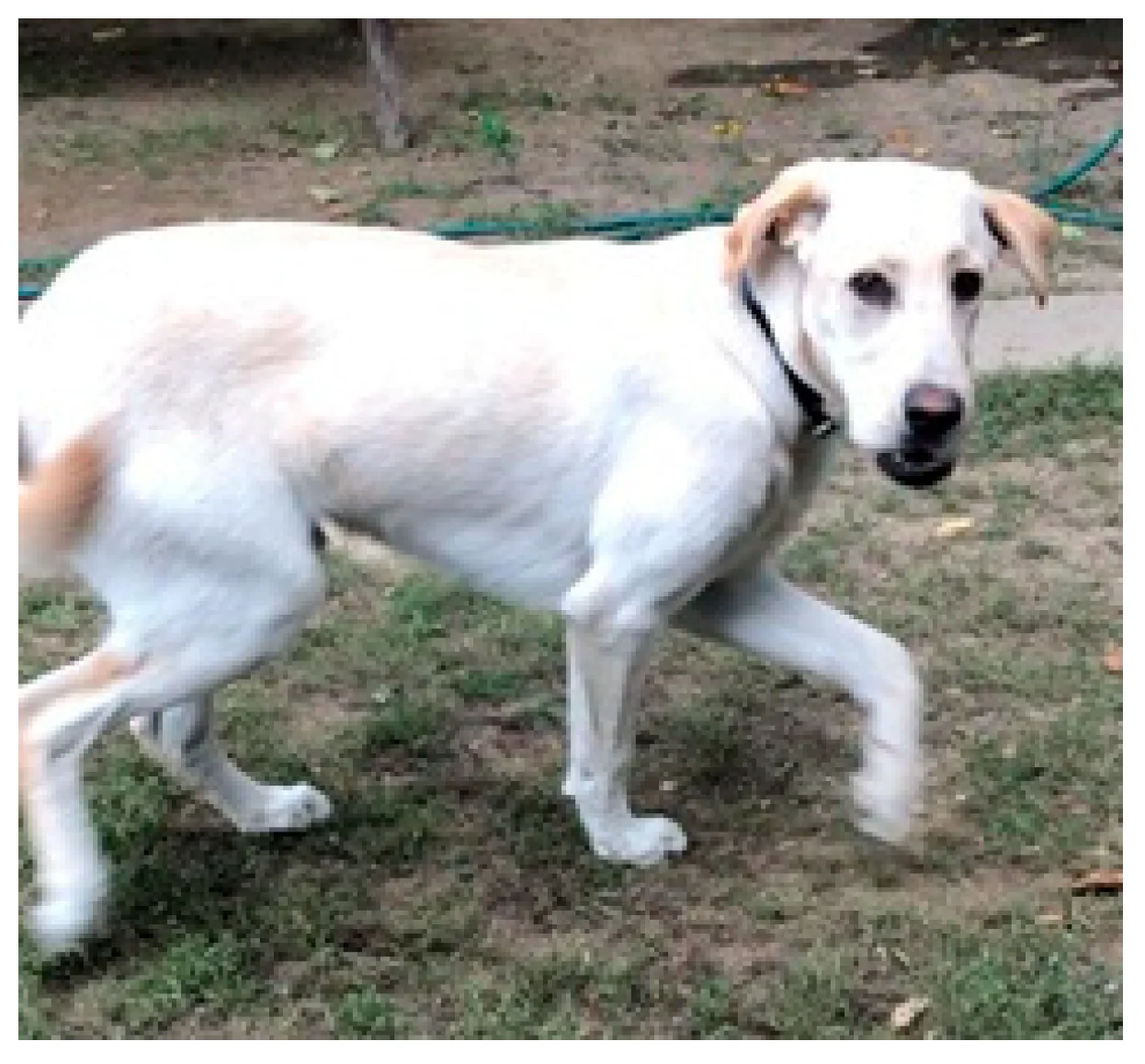
Four-month-old Labrador Retriever with generalized muscular rigidity of the limbs and face and an uncoordinated gait.

**Figure 2:**
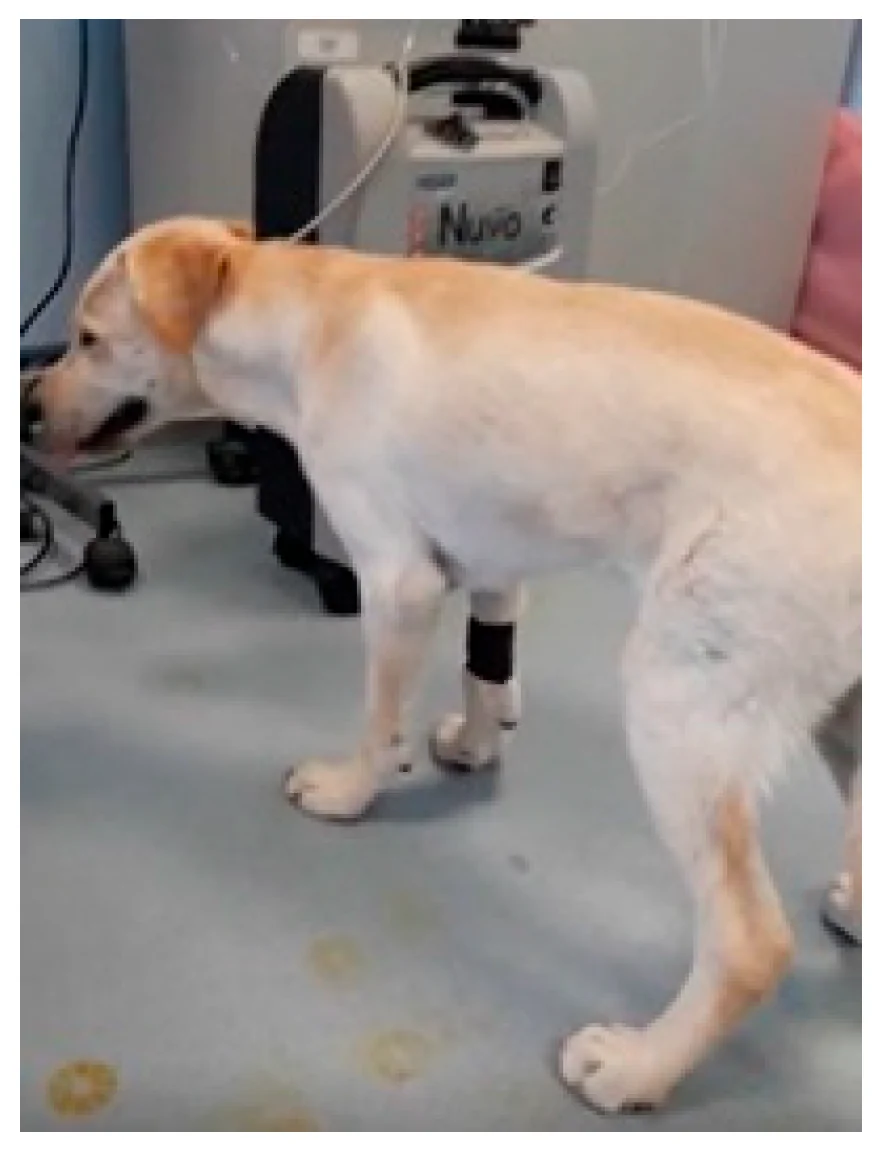
Open-mouth breathing associated with generalized rigidity, hyperthermia and dyspnea.

**Figure 3:**
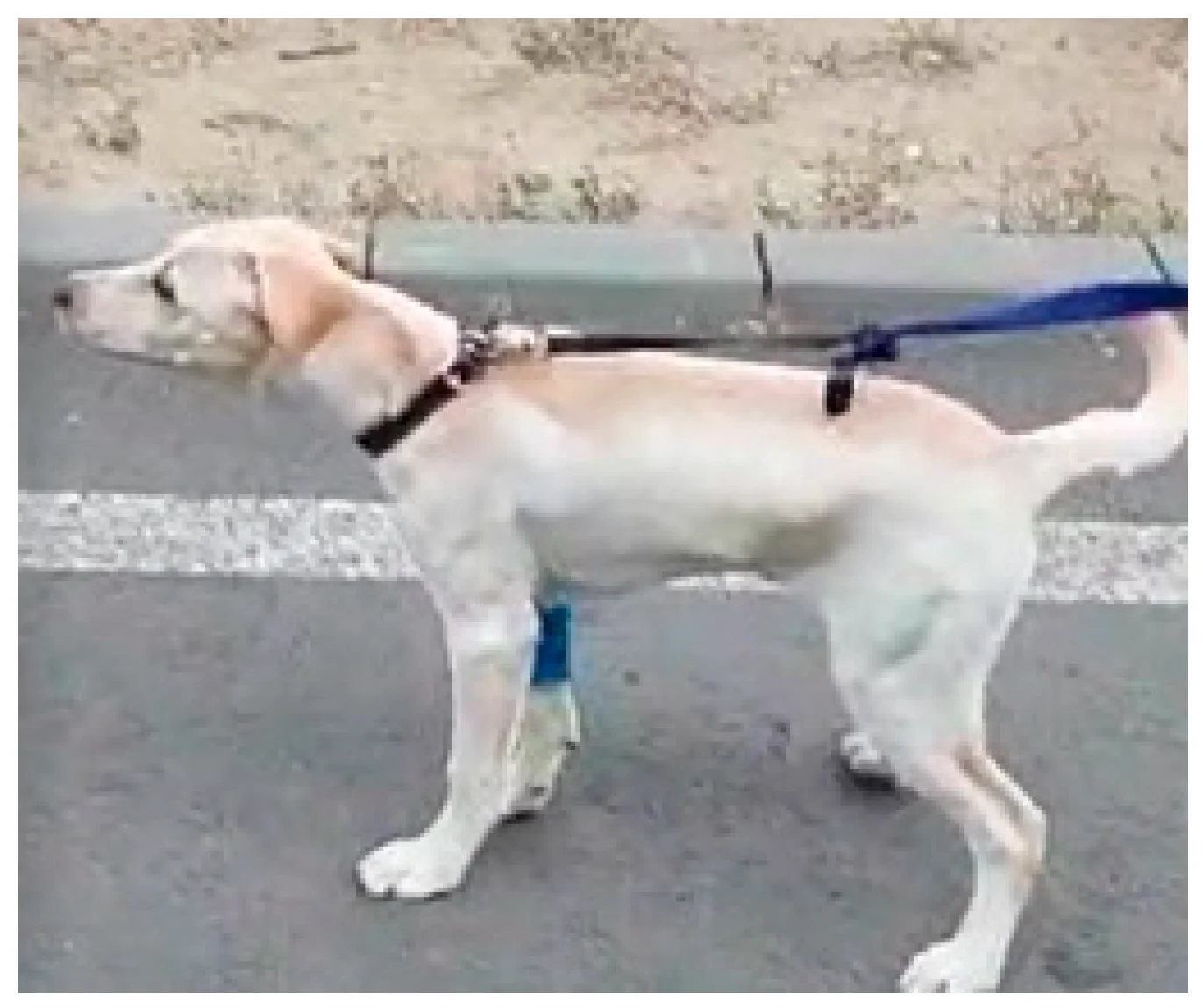
Characteristic “sawhorse” stance.

**Table 1:** Hematological, serum biochemical and venous blood-gas findings at admission. Values outside the reference interval are discussed in the text.

Parameter	Result	Reference Interval
Complete blood count (ProCyte Dx)		
Red blood cells (M/µL)	5.55	5.65–8.87
Hematocrit (%)	33.8	37.3–61.7
Hemoglobin (g/dL)	11.8	13.1–20.5
MCV (fL)	60.9	61.6–73.5
Reticulocytes (K/µL)	14.4	10.0–110.0
White blood cells (K/µL)	8.37	5.05–16.76
Neutrophils (K/µL)	4.80	2.95–11.64
Lymphocytes (K/µL)	2.76	1.05–5.10
Monocytes (K/µL)	0.72	0.16–1.12
Platelets (K/µL)	399	148–484
Serum biochemistry (Catalyst Dx)		
Glucose (mg/dL)	103	77–150
Urea nitrogen (mg/dL)	6	7–29
Creatinine (mg/dL)	0.8	0.3–1.2
Total calcium (mg/dL)	10.0	7.8–12.6
Phosphorus (mg/dL)	5.5	5.1–10.4
Total protein (g/dL)	5.5	4.8–7.2
Albumin (g/dL)	2.8	2.1–3.6
Globulin (g/dL)	2.7	2.3–3.8
ALT (U/L)	53	8–75
ALP (U/L)	97	46–337
GGT (U/L)	3	0–2
Total bilirubin (mg/dL)	0.1	0.0–0.8
Cholesterol (mg/dL)	288	100–400
Amylase (U/L)	477	300–1300
Lipase (U/L)	312	100–1500
Creatine kinase (U/L)	990	99–436
Venous blood gas and electrolytes (VetStat)		
pH	7.37	7.31–7.42
Bicarbonate (mmol/L)	19.7	20.0–29.0
pCO_2_ (mmHg)	37.0	32.0–49.0
Total CO_2_ (mmol/L)	20.8	21.0–31.0
Anion gap (mmol/L)	22	–
Sodium (mmol/L)	155.0	144.0–160.0
Potassium (mmol/L)	5.7	3.5–5.8
Chloride (mmol/L)	119.0	109.0–122.0

MCV—mean corpuscular volume; ALT—alanine aminotransferase; ALP—alkaline phosphatase; GGT—gamma-glutamyl transferase; pCO_2_—partial pressure of carbon dioxide. Reference intervals are those of the analyzers used (adult canine intervals).

## Data Availability

The data supporting the findings of this case report are available from the corresponding authors upon reasonable request.
